# Normalization of network activity in an epilepsy model with a constitutively active *GABBR2* variant

**DOI:** 10.1093/brain/awaf356

**Published:** 2025-09-25

**Authors:** Michal Stawarski, Daniel Ulrich, Sebastian Reinartz, Jochen Schwenk, Li-Yuan Chen, Diego Fernandez-Fernandez, Bartosz Adam Frycz, Mehdi Tafti, Yoon Jeon, Ho Lee, Je Kyung Seong, Murim Choi, Robert Lütjens, Martin Gassmann, Bernd Fakler, Tania Rinaldi Barkat, Bernhard Bettler

**Affiliations:** Department of Biomedicine, Pharmazentrum, University of Basel, Basel CH-4056, Switzerland; Department of Biomedicine, Pharmazentrum, University of Basel, Basel CH-4056, Switzerland; Department of Biomedicine, Pharmazentrum, University of Basel, Basel CH-4056, Switzerland; Institute of Physiology II, University of Freiburg, Freiburg 79104, Germany; Department of Biomedical Sciences, Faculty of Biology and Medicine, University of Lausanne, Lausanne CH-1005, Switzerland; Department of Biomedicine, Pharmazentrum, University of Basel, Basel CH-4056, Switzerland; Department of Biomedicine, Pharmazentrum, University of Basel, Basel CH-4056, Switzerland; Department of Biomedical Sciences, Faculty of Biology and Medicine, University of Lausanne, Lausanne CH-1005, Switzerland; Department of Cancer Biomedical Science, Graduate School of Cancer Science and Policy, National Cancer Center, Gyeonggi 10408, Republic of Korea; Department of Cancer Biomedical Science, Graduate School of Cancer Science and Policy, National Cancer Center, Gyeonggi 10408, Republic of Korea; Korea Mouse Phenotyping Center, College of Veterinary Medicine, Seoul National University, Seoul 08826, Republic of Korea; Department of Biomedical Sciences, Seoul National University College of Medicine, Seoul 03080, Republic of Korea; Addex Therapeutics, Geneva CH-1202, Switzerland; Department of Biomedicine, Pharmazentrum, University of Basel, Basel CH-4056, Switzerland; Institute of Physiology II, University of Freiburg, Freiburg 79104, Germany; Signalling Research Centres BIOSS and CIBSS, University of Freiburg, Freiburg 79104, Germany; Department of Biomedicine, Pharmazentrum, University of Basel, Basel CH-4056, Switzerland; Department of Biomedicine, Pharmazentrum, University of Basel, Basel CH-4056, Switzerland

**Keywords:** GABA_B_ receptors, epileptic encephalopathy, Rett syndrome, autism spectrum disorder, intellectual disability, neurodevelopmental disorders

## Abstract

The neurotransmitter GABA activates G protein-coupled GABA_B_ receptors (GBRs) that mediate neuronal inhibition in the brain. These receptors function as obligate heterodimers, consisting of the GB1 and GB2 subunits, with GB1 binding GABA and GB2 interacting with the G protein. The monoallelic variants p.A567T, p.S695I and p.I705N in the *GABBR2* gene, which encodes the GB2 subunit, have been associated with epileptic encephalopathy and Rett-like disorders. The clinical phenotypes overlap with those seen in individuals with monoallelic loss-of-function variants in *GABBR1*, the gene encoding the GB1 subunit.

To investigate the effects of these *GABBR2* variants on GBR function, we expressed the variants in heterologous cells and evaluated their pharmacological profiles using a luciferase reporter assay. Furthermore, we introduced the epileptic encephalopathy-associated p.I705N variant into the mouse *Gabbr2* gene to examine its impact on neuronal and network activity. These mice were analysed using proteomic approaches, in combination with *in vitro* and *in vivo* electrophysiological techniques. Finally, we evaluated whether the observed network alterations could be reversed pharmacologically.

In heterologous cells, all variants displayed strong constitutive activity, reaching 50%–100% of the maximal GABA-induced activity of wild-type receptors. This gain-of-function effect was evident regardless of whether the variants were expressed as individual subunits or as heterodimeric receptors. EEG recordings from *Gabbr2*^I704N/+^ mice revealed abnormal high-amplitude synchronization in the δ frequency band, without overt seizures. Electrophysiological recordings from brain slices confirmed an increase in constitutive activity in both pre- and postsynaptic GBRs, but also revealed a significant reduction in receptor responsiveness to agonists. Proteomic analysis of brain tissue further revealed a downregulation of both GB1 and GB2 subunits, along with several G protein signalling components. This downregulation likely serves as an adaptive response to the heightened constitutive activity, reducing not only the activity of the variant receptors but also the signalling of wild-type receptors. *In vivo* recordings from the auditory cortex of awake *Gabbr2*^I704N/+^ mice revealed reduced spontaneous neuronal activity and a slower decline in neuronal activity following auditory stimuli. Treatment with a positive allosteric modulator of GBRs normalized spontaneous network activity and the termination of neuronal activity after sensory stimulation in these mice.

In conclusion, our findings indicate that the clinical phenotypes associated with constitutively active *GABBR2* variants are driven by an adaptive downregulation of GBRs and their key signalling components. Therefore, in monoallelic individuals, positive allosteric modulators that enhance wild-type receptor activity may provide a promising therapeutic strategy.

## Teaser

Stawarski *et al*. studied GABA-B receptor gene variants associated with epilepsy and Rett-like syndromes and found that these variants increase constitutive receptor activity, leading to downregulation of brain signalling components. Modulating receptor activity restored network activity, suggesting a promising treatment approach.

## Introduction

Synaptic inhibition is essential for maintaining homeostasis and controlling neuronal network activity.^1^ The main inhibitory neurotransmitter in the mammalian brain, GABA, acts through GABA_A_ and GABA_B_ receptors (GBRs). While GABA_A_ receptors are chloride channels that generate fast synaptic inhibition, GBRs are G protein-coupled receptors that induce pre- and postsynaptic inhibition by gating Cav-type Ca^2+^ and Kir3-type K^+^ channels.^2,3^ GBRs consist of GB1 and GB2 subunits, neither of which is functional on its own.^4^ It is well-established that GABA binding to GB1 induces conformational changes that activate the G protein at GB2.^5-11^ The kinetics of the G protein response are further regulated by the auxiliary GBR subunits KCTD8, −12, −12b and −16,^12-14^ as well as regulators of G protein signalling (RGS) proteins.^15^

GBRs have long been implicated in neurological and psychiatric disorders.^1-3,16^ Recent genetic studies have directly linked variants in *GABBR1* and *GABBR2*, the genes encoding the GB1 and GB2 subunits, with such disorders. Four monoallelic missense variants in *GABBR1*, leading to a partial or complete loss-of-function, result in neurodevelopmental disorders characterized by motor and/or language delay, autism spectrum disorder and in some cases epilepsy.^17,18^ These conditions likely arise from an elevated excitatory-inhibitory (E-I) ratio in neuronal networks.^1,17^ Similarly, monoallelic missense variants in *GABBR2,* such as p.T94M, p.R212Q, p.R212W, p.G440R, p.M668L, p.Y691S, p.G693W, p.S695I and p.I705N, have been linked to neurodevelopmental delay and epileptic encephalopathy (DEE59, OMIM 617904).^18-27^ Clinical symptoms of these variants include infantile spasms with hypsarrhythmia in the EEG, seizures, intellectual disability, absent language, autism spectrum disorder, hand stereotypies, sleep disturbances and generalized hypotonia with paroxysmal limb dystonia. Additionally, the *GABBR2* missense variants p.A567T and p.A707T are associated with a Rett-like phenotype termed neurodevelopmental disorder with poor language and loss of hand skills (NDPLHS, OMIM 617903), occasionally accompanied by seizures.^18,21,28-34^ The clinical similarities between *GABBR1* and *GABBR2* variants, including the emergence of epilepsy, suggest that *GABBR2* variants, like the characterized *GABBR1* variants,^17^ disrupt GBR function. Supporting this hypothesis, a recent study in transfected cultured neurons found that the *GABBR2* p.G693W, p.S695I and p.I705N variants impair GBR surface expression, leading to a presynaptic loss-of-function.^24^ Conversely, another *in vitro* study using heterologous cells showed that the p.I705N and p.A567T variants exhibit constitutive GBR activity, indicating a gain-of-function.^29^ The pathogenic mechanism of *GABBR2* variants therefore remains unclear.

To causally link monoallelic *GABBR2* variants to neuronal dysfunction and disease, mouse models that preserve endogenous expression of both the wild-type (wt) and variant alleles are most informative. Thus, we generated *Gabbr2*^I704N/+^ mice carrying the p.I705N variant in the mouse gene. Electrophysiology in brain slices revealed significantly increased constitutive activity at pre- and postsynaptic GBRs, consistent with the increased constitutive activity observed for the p.I705N variant in transfected non-neuronal cells. Proteomic analysis revealed that *Gabbr2*^I704N/+^ mice adapt to constitutive activity by downregulating GB1, GB2 and key GBR signalling proteins, including G protein subunits. This downregulation also impairs wt receptor signalling and, along with reduced GABA efficacy at the variant receptor, leads to diminished agonist-induced GBR responses. Consistent with the impaired synaptic GBR function, *Gabbr2*^I704N/+^ mice show increased EEG synchronization and delayed network silencing after sensory stimuli. Impaired GBR signalling in response to synaptic GABA may explain why constitutively active *GABBR2* variants cause clinical features resembling *GABBR1* loss-of-function. While positive allosteric modulators (PAMs) have little effect on GABA signalling at *GABBR2* variant receptors, they enhance synaptic signalling at wt receptors. Notably, the PAM ADX71441^35^ normalized the termination of sensory-evoked network activity in *Gabbr2*^I704N/+^ mice, supporting GBR PAMs as a potential therapy for disorders linked to constitutively active *GABBR2* variants.

## Materials and methods

### Plasmids and reagents

The mGluR5 signal peptide and a Myc-tag were introduced into the plasmids encoding human GB2, GB2-S695I and GB2-A567T published previously.^21^ The plasmid encoding GB2-I705N was generated from Myc-tagged GB2 through site-directed mutagenesis (Q5 Site-Directed Mutagenesis kit, New England Biolabs). GB2(R), GB2(K) and serum responsive element-luciferase (sreLuc) plasmids were as reported.^36,37^ The following antibodies were used: anti-Myc (#sc-40, RRID:AB_627268, Santa Cruz Biotechnology), anti-GB1 (#ab55051, RRID:AB_941703, Abcam), anti-GB2 (#322205, RRID:AB_2620061, Synaptic Systems), anti-mouse AF568 (#A-11004, Invitrogen) and anti-guinea pig AF488 (#A-11073, Invitrogen). GABA (#0344), baclofen (#0417), CGP54626 (#1088), CGP52432 (#1246) and ML297 (#5380) were from Tocris Bioscience, ADX71441 was from Addex Therapeutics,^38^ poly-L-lysine (#P1399) was from Sigma, and lipofectamine 2000 was from Invitrogen.

### Immunocytochemistry

HEK293T cells, transfected with GB1b and/or Myc-GB2 plasmids, were plated on poly-L-lysine-coated glass coverslips (45 000 cells per well). After 2 days, cells were incubated with anti-Myc antibody (1:1000) for 1 h, fixed, permeabilized and then treated overnight at 4°C with anti-GB2 antibody (1:1000). Secondary antibodies (AF568 and AF488; 1:500) were applied for 90-min at room temperature. Imaging was performed using a Zeiss LSM880 confocal microscope equipped with a PLAN APO 63× oil immersion objective. Fiji (ImageJ) was used to measure average fluorescence intensity over individual cells, adjusting for background and calculating Myc-to-GB2 fluorescence intensity ratios.

### SreLuc assay

The assay was performed as described.^17^ Transfected cells were incubated with GABA, ADX71441, CGP54626 or DMSO in Opti-MEM™-GlutaMAX™ (Invitrogen) for 6 h. Luc activity in lysed cells was measured using the Dual-Luciferase^®^ Assay Kit (Promega) and a Tecan Spark^®^ microplate reader (Männedorf).

### Mice

C57BL/6 *GABBR2^I704N/+^* mice were generated using Cre-Lox recombination technology. In the *GABBR2^I704N^*^/+^ allele, exon 15 was replaced with a mutated exon 15 harbouring a single base-pair substitution (ATC-to-AAC).

### Proteomic analysis

Membrane-enriched protein fractions were prepared from frozen brains as described.^39^ Proteins were separated by SDS-PAGE, silver-stained lanes cut into two pieces and in-gel digested with sequencing grade trypsin (Promega). Vacuum-dried peptides were dissolved in 0.5% (v/v) trifluoroacetic acid. Appropriate amounts were loaded onto trap columns (C18 PepMap100, 5 μm particles, Thermo Fisher Scientific) with 0.05% trifluoroacetic acid and analysed by liquid chromatography-tandem mass spectrometry (Q Exactive Orbitrap Mass Spectrometer). The RAW files were converted into peak lists (Mascot generic format, mgf) with ProteoWizard msConvert (https://proteowizard.sourceforge.io/). All peak lists were searched with Mascot Server (Matrix Science Ltd, London, UK) against a database containing all mouse, rat and human entries of the UniProtKB/Swiss-Prot database (peptide mass tolerance ±5 ppm; fragment mass tolerance 0.8 Da). One missed trypsin cleavage and common variable modifications were accepted. Abundances of all proteins detected in eluates were used to calculate abundance ratios (target normalized ratio). Protein abundances were normalized to the sum of all proteins per genotype.

### Whole-cell recordings in HEK293T cells

HEK293T cells were transiently transfected with plasmids encoding human GB1a, GB2, Kir3.1/3.2 concatamers and EGFP, and plated on poly-L-lysine-coated glass coverslips. After 2–3 days, coverslips were transferred to a recording chamber filled with a low-K^+^ solution (mM): 145 NaCl, 4 KCl, 5 HEPES, 5.5 D-glucose, 1 MgCl_2_ and 1.8 CaCl_2_ (pH 7.4). Recordings were performed using borosilicate pipettes filled with K-gluconate-based pipette solution (mM): 150 K-gluconate, 1.1 EGTA, 10 HEPES, 10 Tris-phosphocreatine, 0.3 NaGTP and 4 MgATP (pH 7.2). Cells were held in voltage-clamp mode at −80 mV (no correction for liquid junction potential). Kir3 currents were induced in a high-K^+^ bath solution (mM): 120 NaCl, 25 KCl, 5 HEPES, 5.5 D-glucose, 1 MgCl_2_ and 1.8 CaCl_2_ (pH 7.4). The signal was amplified with Multiclamp 700B (Axon Instruments), digitized with a Digidata 1440A (5 kHz sampling rate), low-pass filtered at 1 kHz and analysed offline using pClamp10 (Molecular Devices). Only experiments with stable, low (<10 MΩ) access resistances were included in the analysis. Peak and steady-state currents were measured for each experiment.

### Recordings in hippocampal slices

Mice of either sex (postnatal Day 21–28) were anaesthetized with isoflurane and decapitated. The brain was rapidly removed and immersed in 4°C cold artificial CSF (ACSF, in mM): 92 NMDG-Cl, 30 ${\mathrm{HCO}}_{3}^{-}$, 25 D-glucose, 2.5 KCl, 1.25 NaH_2_PO_4_, 0.5 CaCl_2_, 10 MgSO_4_, 20 HEPES, 2 thiourea, 3 pyruvate and 5 ascorbate (pH 7.4 in 95% O_2_/5% CO_2_). Horizontal slices (300 μm thick) were cut on a vibratome (Leica VT1200S) and kept at 37°C for 30 min. Thereafter, the slices were transferred into standard ACSF (mM): 126 NaCl, 26 NaHCO_3_, 2.5 KCl, 1.25 NaH_2_PO_4_, 10 glucose, 2 CaCl_2_, 1 MgCl_2_, equilibrated with 95% O_2_/5% CO_2_ and kept at room temperature. Individual slices were transferred to a submerged recording chamber and superfused with standard ACSF. Pyramidal cells were morphologically identified through a 60× immersion objective under infrared differential interference contrast videomicroscopy. Whole-cell patch-clamp recordings were performed at a holding potential of −60 mV. The intracellular solution contained (mM): 110 CsCH_3_SO_4_, 15 CsCl, 10 EGTA, 10 HEPES, 10 Tris-phosphokreatine, 4 ATP and 0.1 GTP (pH 7.4). Kir3 currents were recorded at a holding potential of −70 mV with a K-gluconate based intracellular solution at 32°C. Excitatory postsynaptic currents (EPSCs) were evoked by activating Schaffer collaterals with brief current pulses generated by an isolated stimulator via bipolar wire electrodes at 0.1 Hz. Tonic receptor activity was assessed by comparing Kir3 currents or evoked EPSC amplitudes before and after applying the inverse agonist CGP52432 (1 μM). ADX71441 (Addex Therapeutics) was dissolved in DMSO. All drugs were bath applied via the superfusion system. For field recordings, a bipolar concentric platinum electrode (FHC) filled with standard ACSF was placed at the border between CA2 and CA1 and fEPSP were recorded in the CA1 stratum radiatum upon stimulation of the Schaffer collaterals. Following a 20-min baseline recording to ensure the stability of field EPSPs (fEPSPs), long-term potentiation (LTP) was induced with four sequences (10 bursts, 5 pulses at 100 Hz) of theta-burst stimulation with an inter-burst interval of 150 ms and a 15-s interval between each repetition. Following a 3-min pause in stimulation to exclude the recording of post-tetanic potentiation,^40^ basal stimulation was resumed and recorded for a duration of 60 min. Data were recorded for offline analysis in WinLTP 3.0.^41^ LTP levels were determined by averaging the relative responses. The slopes of the fEPSPs were obtained by linear regression over the maximum initial slope points (0.5 ms) following the fibre volley.

### EEG, electromyography and polysomnography in freely behaving mice

EEG/EMG headmounts (Pinnacle Technology) were implanted in 8- to 22-week-old male mice under isoflurane-induced anaesthesia. EMG electrodes were placed on the neck muscles, and EEG electrodes were positioned at the following locations: frontal (+1.5, +1.7), parietal (+3.0, +1.7), common reference (−4.0, −2.0) and ground (+0.0, −2.0). The EEG electrode coordinates were measured in millimetres relative to bregma. Recordings began 2 weeks post-surgery at a 2 kHz sampling rate (Sirenia Acquisition, Pinnacle Technology). The EEG signal was downsampled to 200 Hz for power spectral density (PSD) analysis and normalization, following established procedures.^42^ Offline sleep scoring, conducted between Zeitgeber Time (ZT) 6–10, adhered to established protocols.^42,43^

### Primary auditory cortex recordings in awake mice

Animal procedures were approved by the Veterinary Office of Basel-Stadt, Switzerland (license No. 3004–34045). Eight- to 10-week-old male mice (*Gabbr2*^+/+^: *n* = 3; *Gabbr2*^I704N/+^: n = 3) were anaesthetized with isoflurane (4% induction, 1.5%–2.5% surgery). A perfusion chamber was created above the right auditory cortex and a post fixed on the skull of the left hemisphere.^44^ In extracellular recordings, primary auditory cortex (A1) was identified based on functional tonotopy.^45^ Extracellular electrodes with 32 channels (A4 × 8–5 mm-50-200-177-A32, Neuronexus) were inserted orthogonally to the brain surface at a constant depth (electrode tip at 720 ± 160 µm from pia). Sorted units were identified from raw voltage traces using KiloSort version 2 and manually corrected with phy (CortexLab, UCL, London, UK). Clusters classified as single or multiunit were used. Further analysis was performed in MATLAB (Mathworks, MA, USA). Pairwise correlations (Pearson) in A1 neuronal spike count were calculated between neuron pairs (*z*-scored to normalize for firing rate) within each recording session and then pooled across recording sessions.^46^ The durations of synchronous events were calculated following event detection (threshold: median ± standard deviation) from the population spontaneous firing rate (bin size: 20 ms, smoothing: 80 ms) of each recording session. A1 neuronal activity features were evaluated, focusing on tone-onset neurons (selection criterion: 50% of peak response reached within 45 ms after stimulus onset). Putative parvalbumin expressing neurons (classification based on averaged spike waveform parameters of individual units: peak to trough <0.7 ms and peak half-width <0.4 ms) were excluded from the analysis to reduce variability. Individual neuron peri-stimulus time histograms (PSTHs; average across 150 trials) were baseline-subtracted and the averaged population PSTHs were peak normalized. To quantify the parameters of population response dynamics to white noise stimulation, the population PSTH of each set of neurons was parametrized by fitting an exponential decay function peak to the end of the white noise stimulus. The box plots were generated by resampling (bootstrap) the original data set to create 1000 sets of statistically comparable data, allowing for replacement. The permutation test (1000×) was repeated 10 times to evaluate the averaged-values.^47^

### Statistical analysis

Statistical analysis employed Prism 9 (GraphPad Software, Boston, USA). Sample size in all experiments was based on those of similar experiments in previous studies. To control for confounders, we matched the age and sex of the mice used in the experimental analysis. Samples were randomly allocated to the experimental groups unless they were predefined by the genotype. Masking was not performed for any experiment. Data are presented as mean ± standard error of the mean (SEM) unless indicated otherwise. Normality was assessed using the Shapiro-Wilk test, and homoscedasticity with Bartlett and F-tests. Outliers were identified via the median absolute deviation with a ±3 threshold (auditory cortex recordings). Otherwise, no outliers were removed. The statistical tests adopted are described in the figure legends. All *P-*values reported are two-tailed, and a *P-*value of <0.05 was considered statistically significant.

## Results

### GB2 variants increase constitutive GBR activity and reduce maximal efficacy of GABA

**Figure 1:**
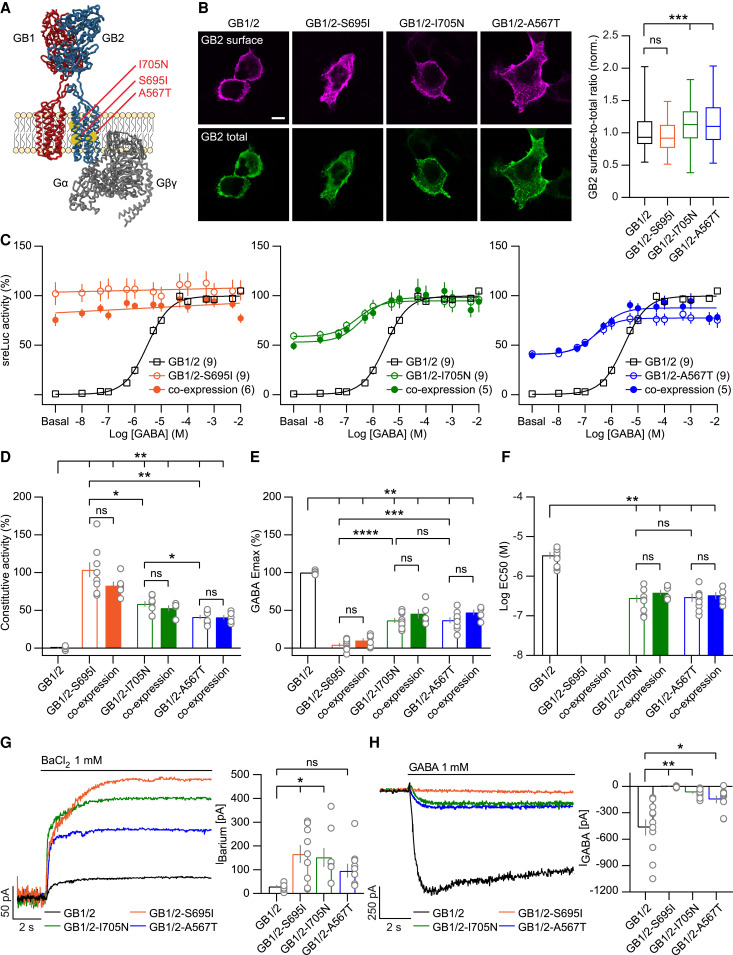
**GB2 variants increase constitutive GBR activity and reduce maximal GABA efficacy**. (**A**) Position of the GB2-S695I, GB2-I705N and GB2-A567T variants in the heterodimeric GB1/2 receptors (PDB: 7EB2). The GB1 (red), GB2 (blue) and intracellular G protein α and βγ subunits (grey) are indicated. (**B**) Cell surface and total Myc-GB2 expression of wt and variant receptors in transfected HEK293T cells. GB1 and Myc-GB2 subunits were co-expressed and the cell surface GB2 immunofluorescence assessed in living cells using an anti-Myc antibody. Total GB2 immunofluroescence was determined with an anti-GB2 antibody after fixation and permeabilization of cells. Scale bar = 10 µm. The box plot shows Myc-GB2 surface-to-total expression ratios for wild-type (wt) GB1/2 (*n* = 148 cells), GB1/2-S695I (*n* = 99), GB1/2-I705N (*n* = 132) and GB1/2-A567T (*n* = 128) receptors. Data are normalized to the GB2 ratio for wt receptors. (**C**) Concentration-response curves of GABA-induced sreLuc activity of wt and variant receptors and a 1:1 mixture of wt and variant receptors. Data are normalized to the maximal sreLuc activity of wt receptors. The GABA concentration-response curve for wt GB1/2 receptors is shown together with those of variant receptors for comparison. The number of independent experiments is indicated. (**D–F**) The bar graph shows the quantitative analysis of constitutive activity (**D**), GABA E_max_ (**E**) and EC_50_ values (**F**) derived from the GABA concentration-response curves shown in **C**. (**G**) Outward currents evoked by BaCl_2_ (*I*_Barium_) in HEK293T cells expressing wt and variant receptors together with Kir3 channels under conditions of high extracellular K^+^ (25 mM) and voltage clamp at −80 mV. The bar graph shows *I*_Barium._ for wt GB1/2 (*n* = 7), GB1/2-S695I (*n* = 9), GB1/2-I705N (*n* = 8), GB1/2-A567T (*n* = 8) receptors (**H**) GABA evoked inward currents (*I*_GABA_) at wt GB1/2 (*n* = 11), GB1/2-S695I (*n* = 8), GB1/2-I705N (*n* = 12) and GB1/2-A567T (*n* = 9) receptors recorded under the same conditions as in (**G**). Data are shown as box plot (median, interquartile range, minimum, maximum) or mean ± standard error of the mean. Kruskal-Wallis with Dunn's *post hoc* (surface-to-total ratio), Welch's ANOVA with Dunnett's T3 *post hoc* (constitutive activity, EC50, *I*_GABA_), one-way ANOVA with Holm-Šidák's *post hoc* (GABA E_max_) or Dunnett's *post hoc* (*I*_Barium_) tests. *P < 0.05, **P < 0.01, ***P < 0.001, ****P < 0.0001, ^ns^*P* > 0.05. E_max_ = maximal GABA-induced response.

The *GABBR2* p.A567T variant localizes to transmembrane domain 3 (TM3), while the p.S695I and p.I705N variants localize to TM6 of the GB2 subunit (Fig. 1A). The A567T variant in TM3 is situated in a region regulating constitutive receptor activity, whereas the S695I and I705N variants in TM6 are located in a region controlling receptor activation and allosteric modulation.^48^ We co-expressed Myc-tagged GB2 variants along with GB1 in HEK293T cells and assessed GB2 cell surface and total expression levels before and after cell permeabilization, respectively, using anti-Myc and anti-GB2 antibodies (Fig. 1B). While GB2-S695I cell surface expression was similar to that of wt GB2, surface expressions of GB2-A567T and GB2-I705N were slightly but significantly increased (Fig. 1B). We generated GABA concentration-response curves using an assay coupling GBRs via chimeric Gα_qi_ to phospholipase C, monitored using a sreLuc reporter.^17,21^ Heterodimeric GB1/2-S695I receptors exhibited full activity in the absence of GABA, reaching 100% of the maximal GABA-induced response (E_max_) observed with wt GB1/2 receptors (Fig. 1C and D). The GB1/2-I705N and GB1/2-A567T receptors showed approximately half of the constitutive activity observed in the GB1/2-S695I receptor, respectively, consistent with previous data indicating constitutive activity for these receptors (Fig. 1C and D).^29^ The wt GB1/2 receptor did not display any constitutive activity in the sreLuc assay (Fig. 1C and D). GABA failed to induce a response in the GB1/2-S695I receptor and induced only 37% of the maximal response observed with the wt GB1/2 receptor in GB1/2-I705N and GB1/2-A567T receptors (Fig. 1C and E). Constitutive activity thus limits the additional response that GABA can elicit. However, the accumulation-based nature of the sreLuc assay may introduce a ceiling effect, potentially leading to an underestimation of E_max_ and, consequently, an overestimation of constitutive activity. In our recombinant assay system, the high receptor density may further amplify constitutive GBR activity. We therefore expect constitutive activity *in vivo*—where receptor expression levels are lower—to be substantially smaller. The EC_50_ values for GABA at GB1/2-I705N and GB1/2-A567T receptors are approximately 10-fold lower than the EC_50_ at wt GB1/2 receptors (Fig. 1C and F), as previously observed with other constitutively active G protein-coupled receptors (GPCRs).^49^ The GABA concentration-response curves and pharmacological profiles obtained from mixed populations of wt and variant receptors, simulating the monoallelic patient scenario, were similar to those of the variant receptors alone (Fig. 1C–F). This finding is unexpected and may indicate a dominant effect of the variant receptors. Constitutively active GB2 variants stabilize the active conformation of the transmembrane domains (TMDs),^11^ which could promote preferential coupling of G proteins to the variant receptors.^10^ However, since the relative abundance of variant and wt receptors at the plasma membrane in our assay system is unknown, their individual contributions to G protein activation in mixed populations cannot be determined. Moreover, GABA-induced signalling may reach saturation in the sreLuc assay, potentially obscuring differences in receptor activity.

We addressed whether GBR variants also constitutively activate Gβγ signalling to Kir3 channels. We blocked K^+^ currents with BaCl_2_ in HEK293T cells co-expressing receptors and Kir3 channels (Fig. 1G). GBR variants displayed a 3 to 6-fold increase in Ba^2+^-blocked current (*I*_Barium_) compared to wt GB1/2 receptors, confirming increased constitutive Kir3 channel activity (Fig. 1G). As a result of this constitutive activity, K^+^ currents activated by a saturating concentration of GABA (*I*_GABA_) were 7- and 3-fold smaller for GB1/2-I705N and GB1/2-A567T receptors, respectively, compared to wt GB1/2 receptors (Fig. 1H). GABA failed to induce a current in fully constitutively active GB1/2-S695I receptors, as previously reported.^24^

### GB2 variants exhibit high constitutive activity in the absence of GB1

**Figure 2:**
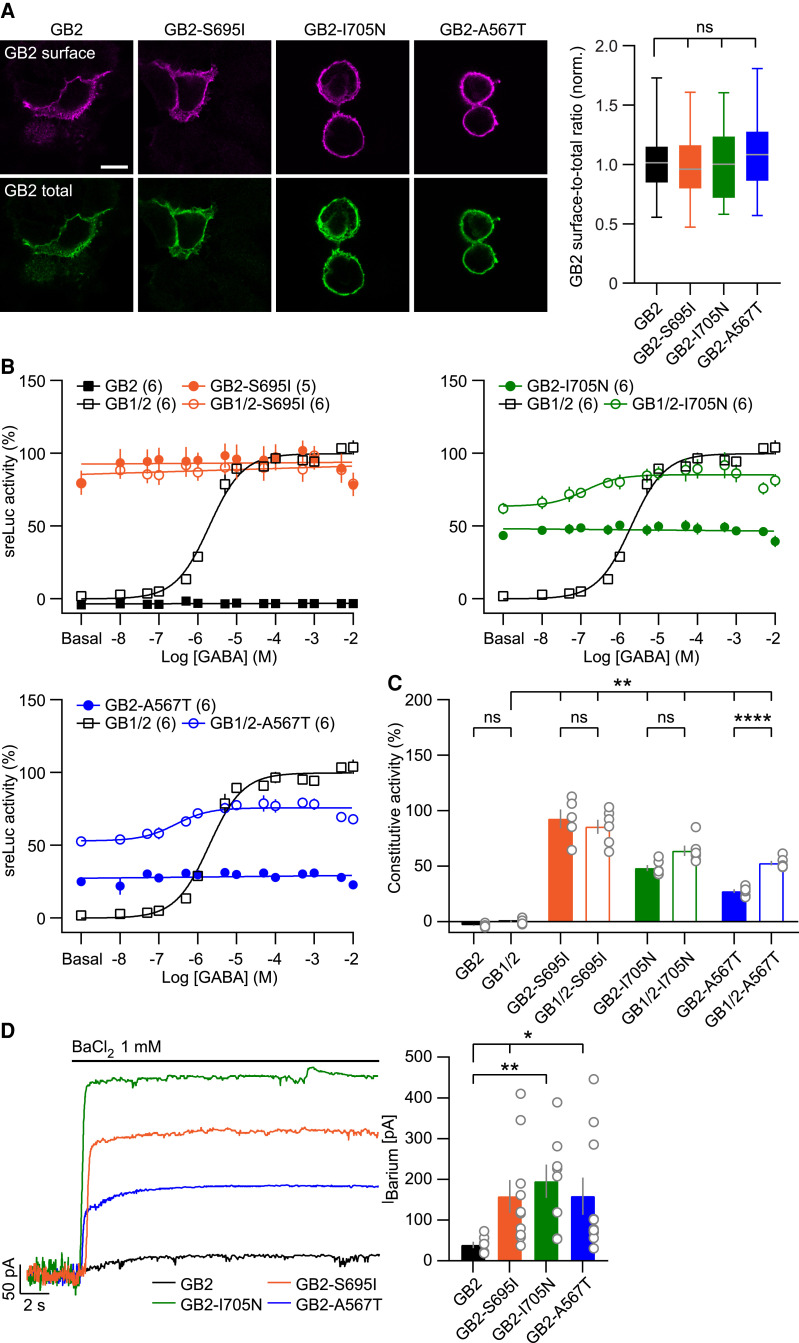
**GB2 subunit variants are constitutively active in the absence of GB1.** (**A**) Cell surface and total Myc-GB2 expression of wild-type (wt) GB2 and GB2 subunit variants expressed in transiently transfected HEK293T cells. Cell surface GB2 immunofluorescence was assessed in living cells using an anti-Myc antibody. Total GB2 immunofluorescence was determined with an anti-GB2 antibody after fixation and permeabilization of cells. Scale bar = 10 µm. The box plot shows surface-to-total expression ratios for wt GB2 (*n* = 73 cells), GB2-S695I (*n* = 73), GB2-I705N (*n* = 73) and GB2-A567T (*n* = 62). Data are normalized to the surface-to-total expression ratio of wt GB2. (**B**) Concentration-response curves of GABA-induced sreLuc activity of variant GB2 subunits and receptors. Data are normalized to the maximal sreLuc activity of wt GB1/2 receptors. All GABA concentration-response curves were determined in parallel. The number of independent experiments is indicated. (**C**) Bar graph of the constitutive activity from the experiments depicted in **B**. Data are normalized to the E_max_ of GABA at wt GB1/2 receptors. (**D**) Outward currents evoked by BaCl_2_ (*I*_Barium_) in HEK293T cells expressing homomeric GB2 variants together with Kir3 channels. Bar graphs show *I*_Barium_ for wt GB2 (*n* = 7 cells), GB2-S695I (*n* = 10), GB2-I705N (*n* = 8) and GB2-A567T (*n* = 10). Data are shown as box plot (median, interquartile range, minimum, maximum) or mean ± standard error of the mean. Kruskal-Wallis with Dunn's *post hoc* (surface-total ratio, *I*_Barium_), Welch's ANOVA with Dunnett's T3 *post hoc* (constitutive activity) tests. *P < 0.05, **P < 0.01, ****P < 0.0001, ^ns^*P* > 0.05. E_max_ = maximal GABA-induced response.

Since G protein activation occurs at the GB2 subunit of the GBR,^2-4^ it is conceivable that GB2 variants might exhibit constitutive activity on their own. In the absence of GB1, GB2 variants traffic similarly to wt GB2 subunits to the plasma membrane in transfected HEK293T cells (Fig. 2A). In the sreLuc assay, wt GB2 alone was inactive, even at saturating GABA concentrations (Fig. 2B and C), indicating that the HEK293T cells used do not express detectable levels of endogenous GB1 capable of forming functional receptors with GB2. In contrast, GB2-S695I alone was fully active (Fig. 2B and C), demonstrating that homomeric GB2-S695I is constitutively active. Homomeric GB2-I705N and GB2-A567T showed reduced constitutive activity compared to GB2-S695I (Fig. 2B and C). Co-transfection of GB1 increased constitutive activity for GB2-I705N and GB2-A567T, but not GB2-S695I; however, the increase was statistically significant only for GB2-A567T (Fig. 2B and C). This increase in constitutive activity in the presence of GB1 was blocked by the inverse agonist CGP54626,^48,50,51^ which binds to the orthosteric site of GB1 (Supplementary Fig. 1A and B), indicating that heteromeric receptors contribute to the overall constitutive activity of these GB2 variants. As expected, CGP54626 failed to block the constitutive activity of GB2 variants expressed in in the absence of GB1 (Supplementary Fig. 1A and B). When surface expression of GB2 subunits was restricted by introducing an artificial ER retention signal,^36^ the resulting GB2(K)-S695I, GB2(K)-I705N and GB2(K)-A567T variants were inactive in the sreLuc assay (Supplementary Fig. 2A–C). These findings underscore the requirement for cell surface expression in G protein activation by GB2 variants. Notably, CGP54626 fully inhibited the constitutive activity of heteromeric receptors assembled with GB2(K)-I705N or GB2(K)-A567T, but not those containing GB2(K)-S695I (Supplementary Fig. 2C and D). This finding suggests that the GB2-S695I variant stabilizes the active receptor interface to a degree that cannot be overcome by CGP54626.^11^ Consistent with the sreLuc assay results, homomeric GB2-S695I, GB2-I705N and GB2-A567T subunits constitutively activated Kir3 channels in transfected HEK293T cells, whereas wt GB2 subunits did not (Fig. 2D). This constitutive Kir3 activation further supports that these homomeric GB2 variants signal from the plasma membrane rather than from intracellular compartments.

### ADX71441 predominantly has PAM activity at wt GBRs

Since constitutive GBR activity negatively impacts synaptic GBR activation, PAMs that enhance GBR signalling may offer therapeutic benefits. The PAM ADX71441^35,38^ significantly increased GABA potency in wt, but not variant, GBRs in the sreLuc assay in transfected HEK293T cells (Supplementary Fig. 3A and B). Additionally, ADX71441 exhibited significant allosteric agonism at both wt and variant receptors, except for the fully constitutively active GB1/2-S695I receptor (Supplementary Fig. 3A and B). Overall, the data show that ADX71441 predominantly exerts PAM effects through the wt GBR. The binding site of ADX71441 has not yet been characterized. However, two chemically distinct PAMs—GS39783 and BHFF—have been shown to bind to overlapping sites buried within the TMD, involving residues from TM5, TM6 and TM7 of both protomers.^5,8,52^ Since ADX71441 did not enhance the constitutive activity of homomeric GB2 variants (Supplementary Fig. 3C), but exhibited significant ago-PAM activity at heteromeric receptor variants (Supplementary Fig. 3A and B), it is likely that ADX71441 binds to the same allosteric pocket at the TMD interface of the GB1/GB2 heterodimer. Nevertheless, the active-state conformation of GBRs may vary slightly depending on the specific combination of agonist and PAM.^9^

### High amplitude δ rhythm in the EEG of *Gabbr2*^I704N/+^ and *Gabbr2*^I704N/I704N^ mice

**Figure 3:**
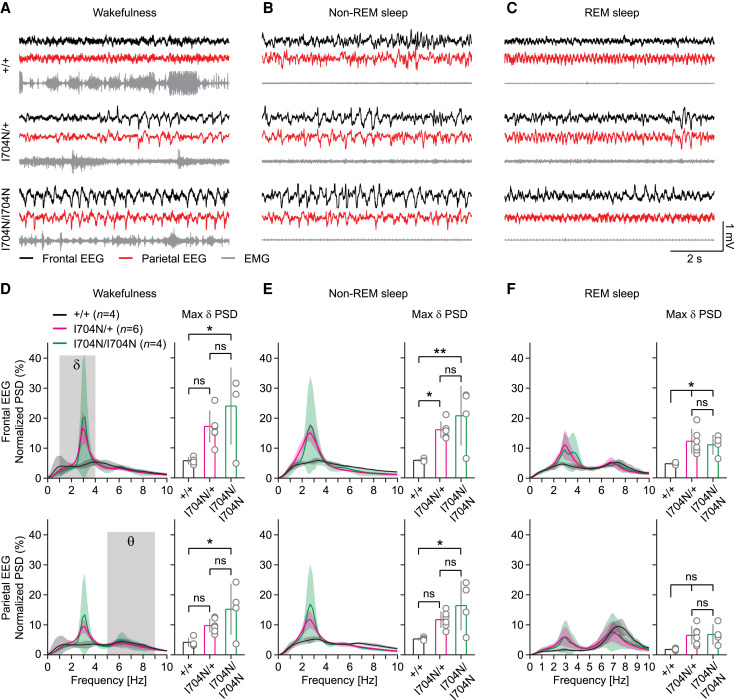
***Gabbr2*^I704N/+^ and *Gabbr2*^I704N/I704N^ mice exhibit a high amplitude δ rhythm in the EEG.** (**A–C**) *In vivo* frontal and parietal EEG with EMG recordings obtained during wakefulness (**A**), non-REM sleep (**B**) and REM sleep (**C**) of *Gabbr2^+/+^* (*n* = 4), *Gabbr2*^I704N/+^ (*n* = 6) and *Gabbr2*^I704N/I704N^ (*n* = 4) mice. For visualization, all traces were down-sampled to 200 Hz and high-pass filtered from 0.7 Hz for EEG and 10 Hz for EMG traces. (**D–F**) EEG PSD profiles and maximum PSDs in the δ (1–4 Hz) frequency bands during wakefulness (**D**), non-REM sleep (**E**) and REM sleep (**F**). All data are mean ± standard error of the mean. One-way ANOVA with Tukey-Kramer *post hoc* tests. *P < 0.05, **P < 0.01, ^ns^*P* > 0.05. EMG = electromyography; REM = rapid eye movement; PSD = power spectral density.

Variants that enhance constitutive activity also exhibit a concurrent reduction in GABA efficacy. The increased constitutive activity represents a gain-of-function, whereas the reduced GABA efficacy constitutes a loss-of-function. This dual effect makes it challenging to predict the impact of these variants on neuronal activity in the nervous system. To address this, we generated *Gabbr2*^I704N/+^ and *Gabbr2*^I704N/I704N^ mice using Cre-Lox recombination technology. These mice carry the *Gabbr2* p.I704N allele, which is homologous to the human p.I705N allele identified in a DEE59 patient.^19,20^ We analysed *Gabbr2*^I704N/+^ and *Gabbr2*^I704N/I704N^ mice for potential EEG abnormalities in the frontal and parietal regions. In control *Gabbr2^+/+^* mice, EEG and EMG activity during wakefulness, non-REM sleep and REM sleep (Fig. 3A–F) were consistent with previous reports.^53^ In contrast, during wakefulness, *Gabbr2*^I704N/+^ and *Gabbr2*^I704N/I704N^ mice exhibited a high-amplitude rhythm in the δ (1–4 Hz) frequency band, which occurred spontaneously in both the frontal and parietal regions (Fig. 3A). To further investigate this δ rhythm, we computed power spectral density (PSD) profiles using a short-time Fourier transform with a Hamming window. Due to the large amplitude of the δ rhythm, the spectrograms showed an increase in the normalized PSD at its harmonic frequencies (Fig. 3D). The duration of this EEG rhythm was variable; although it was sometimes transient, it consistently occurred synchronously between the frontal and parietal regions. Non-REM sleep EEG activity in the frontal and parietal regions of *Gabbr2*^I704N/+^ and *Gabbr2*^I704N/I704N^ mice was almost exclusively dominated by the δ rhythm (Fig. 3B and E). During REM sleep in *Gabbr2*^I704N/+^ and *Gabbr2*^I704N/I704N^ mice, the amplitude of the δ rhythm was also increased (Fig. 3C and F). Overall, the EEG data showed that the normalized frontal and parietal PSD profiles of *Gabbr2*^I704N/I704N^ mice peaked around 3 Hz and were significantly higher than those of *Gabbr2^+/+^* mice during wakefulness, non-REM sleep and REM sleep, with the exception of the parietal EEG during REM sleep (Fig. 3D–F). For *Gabbr2*^I704N/+^ mice, the frontal maximum δ PSD profile was significantly higher during non-REM and REM sleep (Fig. 3D–F). Visual inspection of 4-day EEG/EMG recordings of *Gabbr2*^I704N/+^ and *Gabbr2*^I704N/I704N^ mice did not reveal EEG rhythms resembling electrographic tonic-clonic seizures or the spike-and-wave patterns typical of absence seizures.

### Proteomic data reveal adaptive changes in *Gabbr2^I704N/+^* and *Gabbr2^I704N/I704N^* mouse brains

**Figure 4:**
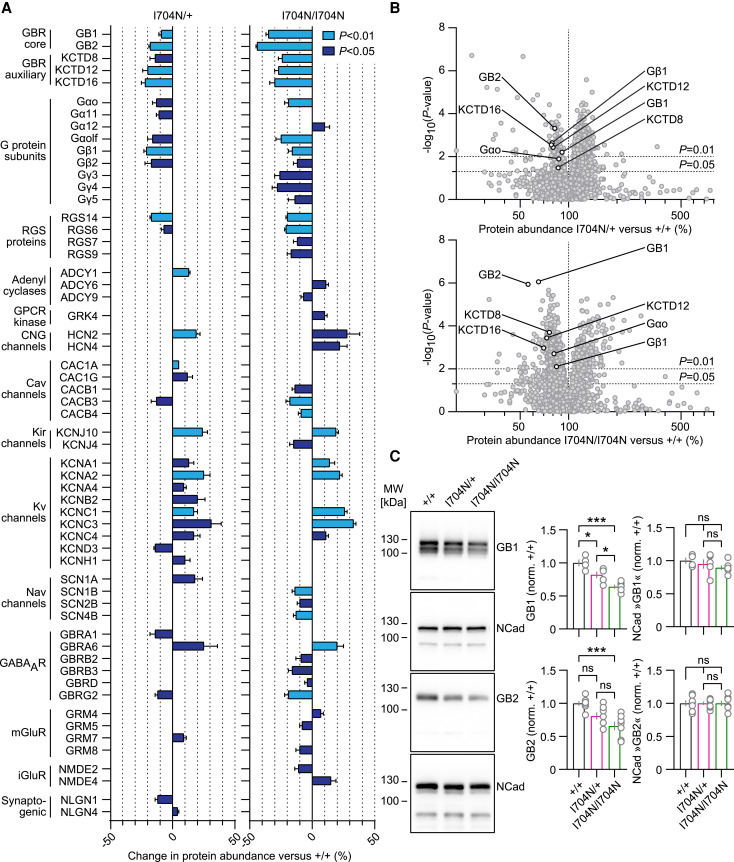
**Homeostatic changes in protein abundances in solubilized brains of *Gabbr2*^I704N/+^ and *Gabbr2*^I704N/I704N^ mice**. (**A**) Altered protein abundances in *Gabbr2*^I704N/+^ and *Gabbr2*^I704N/I704N^ mice versus littermate *Gabbr2^+/+^* mice. Negative values indicate a decrease relative to *Gabbr2^+/+^* brain membranes, while positive values indicate an increase relative to *Gabbr2^+/+^*. Bar graphs are shaded according to the *P*-value: light blue *P* < 0.01, dark blue *P* < 0.05. Five mice per genotype were analysed. (**B**) Scatter plots of log(*P*-values) versus protein abundance (log scale) for all proteins detected in the experiment for *Gabbr2*^I704N/+^ (*top*) and *Gabbr2*^I704N/I704N^ (*bottom*) brain membranes. Vertical line indicates protein abundance equal to *Gabbr2^+/+^*. Horizontal lines indicate *P* = 0.01 and *P* = 0.05 significance thresholds. (**C**) Representative immunoblots and quantification of GB1, GB2 and N-cadherin (NCad) from brain membranes of *Gabbr2^+/+^*, *Gabbr2*^I704N/+^ and *Gabbr2*^I704N/I704N^ mice. Data are normalized to protein expression in *Gabbr2^+/+^* mice. *Gabbr2^+/+^* (GB1 *n* = 4 mice; GB2 *n* = 7), *Gabbr2*^I704N/+^ (GB1 *n* = 5; GB2 *n* = 7) and *Gabbr2*^I704N/I704N^ (GB1 *n* = 6; GB2 *n* = 7). Proteomic analysis: *t*-test; and GB1 and GB2 expression: one-way ANOVA with Tukey's *post hoc* tests. All data are mean ± standard error of the mean. *P < 0.05, ***P < 0.001, ^ns^*P* > 0.05.

Using quantitative mass spectrometry, we studied whether increased constitutive activity leads to adaptive changes in the brain proteome of *Gabbr2*^I704N/+^ and *Gabbr2*^I704N/I704N^ mice. Compared to *Gabbr2*^+/+^ mice, 110 and 429 proteins were downregulated (ratio <0.8, *P* < 0.01), while 260 and 147 proteins were upregulated (ratio >1.2, *P* < 0.01) (Fig. 4A and B). The changes in protein abundance are generally more pronounced in *Gabbr2*^I704N/I704N^ mice than in *Gabbr2*^I704N/+^ mice. Notably, the receptor subunits GB1 and GB2, along with the auxiliary subunits KCTD8, KCTD12 and KCTD16,^2,12,54^ were downregulated in the mutant mice (Fig. 4A and B). This downregulation was further validated for GB1 and GB2 through immunoblot quantification (Fig. 4C). Since GABA activation enhances the rate of GBR internalization,^55^ the reduced protein levels may be due to increased internalization and degradation of the variant receptors as a result of their constitutive activity. Similarly, the G protein subunits Gαo, Gβ1 and Gβ2—key components of the multiprotein GBR signalling complex in the brain^12,14^—were significantly downregulated in the mutant mice. Additionally, other G protein subunits and RGS proteins were downregulated. GBR-linked effector channels^56-58^ also underwent significant adaptive changes: the β subunits of GBR effector Cav channels, were downregulated, while HCN2 channels were upregulated. However, the abundance of Kir3 channels, the primary postsynaptic effector K^+^ channels of GBRs, remained unchanged. Other notable changes in the mutant mice included the upregulation of Kv1 (KCNA), Kv3 (KCNC) and Kir4 (KCNJ10) K^+^ channel subunits, as well as the GABA_A_ receptor subunit α6. These altered protein abundances likely represent adaptations to a disturbed E/I balance in the brain, caused by the elevated constitutive activity of GBRs. Of note, the downregulation of GBR signalling components observed in the presence of constitutively active GBRs may help explain why chronic baclofen administration often leads to receptor desensitization and the development of tolerance.^59^ Interestingly, the downregulation of signalling proteins shared among GPCRs—such as G protein subunits and RGS proteins—may also impair signalling through other GPCRs, potentially contributing to some of the adaptive changes observed.

### Increased tonic and reduced agonist-induced GBR activity in *Gabbr2^I704N/+^* and *Gabbr2^I704N /I704N^* mice

**Figure 5:**
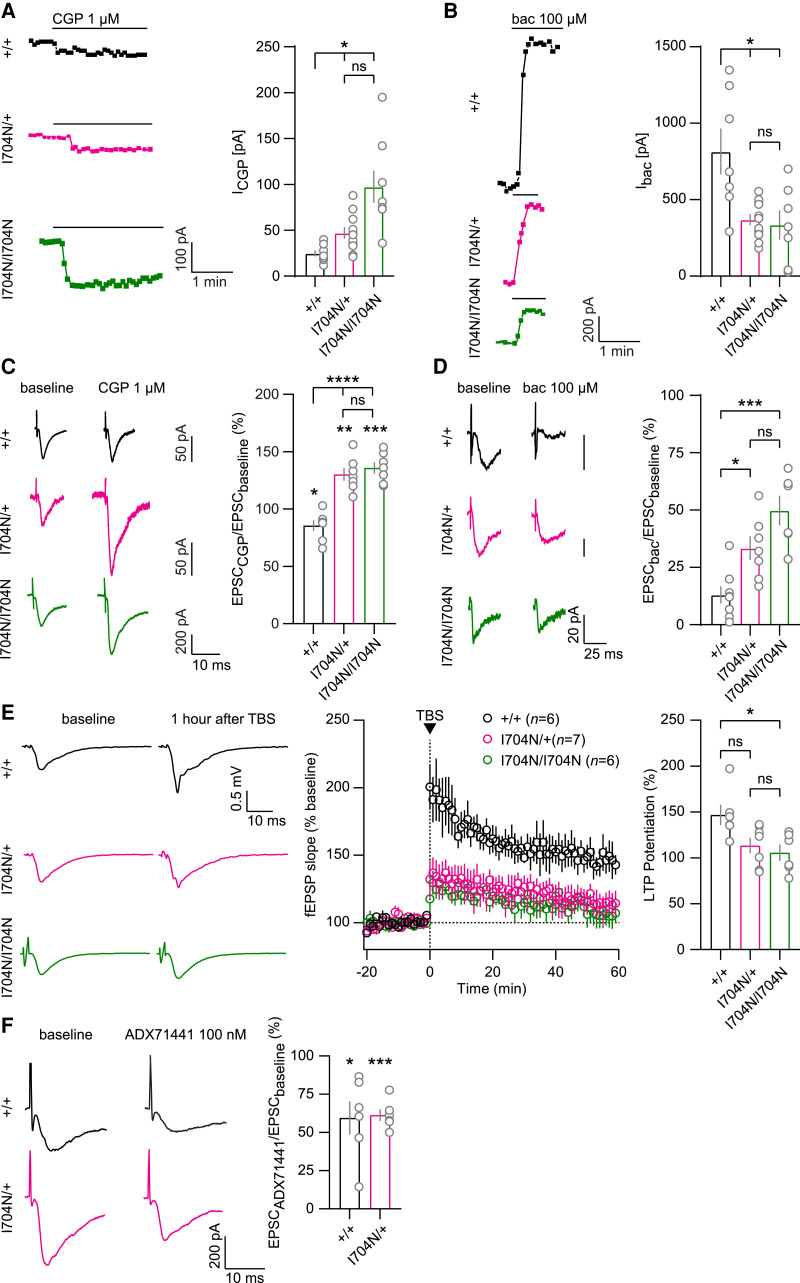
**Increased tonic and reduced baclofen-induced GBR activity in *Gabbr2*^I704N/+^ and *Gabbr2*^I704N/I704N^ mouse brains**. (**A–D** and **F**) Patch-clamp recordings from pyramidal neurons in acute hippocampal slices in neurons of mutant and littermate mice. (**A**) Examples of currents blocked by the GBR inverse agonist CGP52432 (*I*_CGP_, tonic GBR-induced current). Bar graph shows quantification of *I*_CGP52432_ recorded from *Gabbr2^+/+^* (*n* = 8 neurons), *Gabbr2*^I704N/+^ (*n* = 11) and *Gabbr2*^I704N/I704N^ (*n* = 8) hippocampal slices. (**B**) Examples of K^+^ currents induced by baclofen (*I*_Bac_). Bar graph shows quantification of *I*_Bac_ recorded from *Gabbr2^+/+^* (*n* = 7 neurons), *Gabbr2*^I704N/+^ (*n* = 7) and *Gabbr2*^I704N/I704N^ (*n* = 7) slices. (**C**) Examples of CGP52432-mediated augmentation of evoked EPSC amplitudes. Bar graph shows ratio of EPSC amplitudes after versus before CGP52432 application, recorded from *Gabbr2^+/+^* (*n* = 7 neurons), *Gabbr2*^I704N/+^ (*n* = 7) and *Gabbr2*^I704N/I704N^ (*n* = 7) slices. (**D**) Examples of baclofen-mediated inhibition of evoked EPSC amplitudes. Bar graph shows ratio of EPSC amplitudes after versus before baclofen application, recorded from *Gabbr2^+/+^* (*n* = 8 neurons), *Gabbr2*^I704N/+^ (*n* = 7) and *Gabbr2*^I704N/I704N^ (*n* = 6) slices. (**E**) Examples of field EPSPs (fEPSPs) at baseline and 60 min after theta-burst stimulation (TBS; *left*) recorded in acute hippocampal slices. Time course of fEPSP slopes recorded from *Gabbr2^+/+^* (*n* = 6), *Gabbr2*^I704N/+^ (*n* = 7) and *Gabbr2*^I704N/I704N^ (*n* = 6) slices. The arrowhead indicates the time of TBS. The bar graph shows the fEPSP slope 60 min after TBS. Long-term potentiation (LTP) was reduced in the mutant mice. (**F**) Examples of ADX71441-mediated inhibition of evoked EPSC amplitudes in pyramidal neurons. Bar graph shows ratio of EPSC amplitudes after versus before ADX71441 application recorded from *Gabbr2^+/+^* (*n* = 6 neurons) and *Gabbr2*^I704N/+^ (*n* = 6) slices. All data are mean ± standard error of the mean. Welch's ANOVA with Dunnett's T3 *post hoc* (tonic K^+^ currents), one-way ANOVA with Tukey's *post hoc* (*I*_bac_, EPSC_bac_, LTP) or Šidák's *post hoc* (EPSC_CGP52432_). One-sample *t*-test (EPSC_CGP52432_) and (EPSC_ADX71441_). *P < 0.05, **P < 0.01, ***P < 0.001, ****P < 0.0001, ^ns^*P* > 0.05. EPSC = excitatory postsynaptic current.

Proteomic experiments suggest that the increased constitutive activity of the GB2-I705N variant induces adaptations in GBR signalling components, ultimately leading to attenuated GABA-induced receptor signalling. To investigate both constitutive GBR activity and the response to the GBR agonist baclofen in *Gabbr2*^I704N/+^ and *Gabbr2*^I704N/I704N^ mice, we performed patch-clamp recordings on CA1 pyramidal neurons in acute hippocampal slices. Inhibition of GBR activity using the inverse agonist CGP52432^51^ revealed significantly larger tonic K^+^ currents in *Gabbr2*^I704N/+^ and *Gabbr2*^I704N/I704N^ neurons compared to *Gabbr2^+/+^* neurons (Fig. 5A). These findings suggest that constitutively active GBRs assembled with the GB2 variant activate Kir3 channels at the plasma membrane, leading to the observed increased tonic K^+^ currents in the mutant mice. A saturating concentration of baclofen induced significantly smaller K^+^ currents in *Gabbr2*^I704N/+^ and *Gabbr2*^I704N/I704N^ neurons than *Gabbr2^+/+^* neurons (Fig. 5B). Since the proteomic data show no evidence of Kir3 channel downregulation in the mutant mice, the smaller baclofen-induced currents are likely attributable to a downregulation of GBR signalling components (Fig. 4A and B) and a reduction in the GABA efficacy (Fig. 1C and E) at the variant receptors. Presynaptic GBRs suppress neurotransmitter release by inhibiting voltage-sensitive Ca^2+^ channels.^2,3^ Consistent with the presence of constitutively active GBRs assembled with the GB2 variant at the presynaptic plasma membrane, CGP52432 significantly increased the amplitudes of evoked excitatory postsynaptic currents (EPSCs) in *Gabbr2*^I704N/+^ and *Gabbr2*^I704N/I704N^ neurons (Fig. 5C). In contrast, in *Gabbr2^+/+^* neurons, application of CGP52432 led to a slight but significant reduction in EPSC amplitudes. Accordingly, the EPSC_CGP_/EPSC_baseline_ ratio was significantly increased in *Gabbr2*^I704N/+^ and *Gabbr2*^I704N/I704N^ neurons compared to *Gabbr2^+/+^* neurons (Fig. 5C). Baclofen was significantly less efficient in reducing the amplitudes of evoked EPSCs in *Gabbr2*^I704N/+^ and *Gabbr2*^I704N/I704N^ mice, indicating a reduced activation of presynaptic GBRs (Fig. 5D). Overall, our data are consistent with the proteomic and *in vitro* pharmacological findings, revealing increased constitutive and reduced baclofen-induced GBR signalling in *Gabbr2*^I704N/+^ and *Gabbr2*^I704/I704N^ neurons.

LTP is recognized as a cellular mechanism crucial for learning and memory formation.^60^ Previous studies have shown that genetic ablation of GBRs or their pharmacological blockade impairs LTP.^61,62^ We used field recordings to analyse LTP at the Schaffer collateral-commissural to CA1 pyramidal cell synapse in hippocampal slices. Theta-burst stimulation (TBS) elicited robust LTP in *Gabbr2*^+/+^ mice, with an average potentiation of 147% above baseline during the last 5 min of a 60-min recording session. LTP was significantly reduced to 113% and 105% in slices from *Gabbr2*^I704N/+^ and *Gabbr2*^I704N/I704N^ mice, respectively (Fig. 5E). These LTP results are consistent with impaired GBR signalling.

### ADX71441 modulates evoked EPSCs through wt GBRs in *Gabbr2^I704N/+^* mice

The PAM ADX71441^35,38^ significantly increased GABA potency in wt, but not variant, GBRs in the sreLuc assay in transfected HEK293T cells (Supplementary Fig. S3A and B). Additionally, ADX71441 exhibited significant allosteric agonism at both wt and variant receptors, except for the fully constitutively active GB1/2-S695I receptor (Supplementary Fig. S3A and B). Overall, the data show that ADX71441 predominantly exerts positive allosteric effects through the wt GBR. We next investigated whether ADX71441 exhibited allosteric activity in electrophysiological experiments using acute brain slices from *Gabbr2*^I704N/+^ mice. ADX71441 significantly reduced evoked EPSCs in CA1 pyramidal neurons of *Gabbr2*^I704N/+^ mice (Fig. 5F). This finding suggests that PAMs could be beneficial in enhancing synaptic GBR responses through the wt GBR in individuals with constitutively active, monoallelic *GABBR2* variants.

### Allosteric modulation of GBRs normalizes network activity in *Gabbr2^I704N/+^* mice

**Figure 6:**
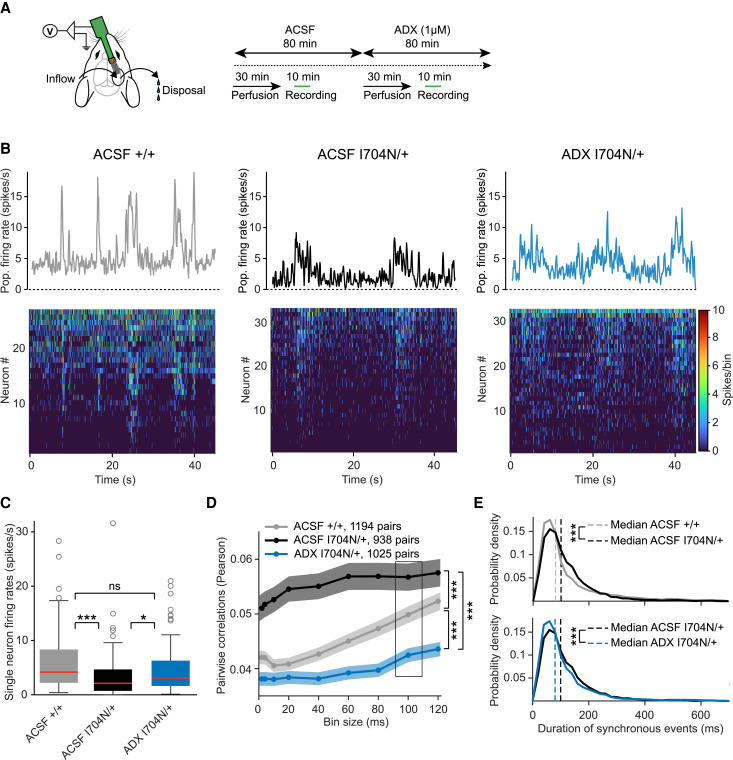
**ADX71441 normalizes spontaneous firing rates and pairwise correlation of neuronal firing in *Gabbr2*^I704N/+^ mouse primary auditory cortex**. (**A**) *Left*: Multi-channel electrophysiological recording (4 shanks, tot. 32 channels) in *A1* (right hemisphere) of a head-fixed, awake mouse positioned in a sound box. Artificial CSF (ACSF) solutions (36°C) were slowly perfused over the cortical surface. *Right*: Experimental flow scheme: Neuronal activity was recorded for 10 consecutive min (green lines) following a 30-min perfusion with either ACSF or 1 µM ADX71441 dissolved in ACSF. (**B**) Examples of spontaneous activity recordings (45 s) from *A1* in a *Gabbr2*^+/+^ mouse perfused with ACSF (*left*) and a *Gabbr2*^I704N/+^ mouse perfused with ACSF (*middle*) or 1 µM ADX71441 (*right*). *Top*: Averaged population firing rate using 100 ms bins with 400 ms smoothing. *Bottom*: Binned (100 ms) activity of individual neurons, sorted according to averaged firing rate. (**C**) Reduced single neuron firing rate in *Gabbr2*^I704N/+^ mice (*n* = 76 neurons, 3 mice, black bar) as compared to *Gabbr2^+/+^* mice (*n* = 86 neurons, 3 mice, grey bar). Significantly increased firing rates in the same neurons of *Gabbr2*^I704N/+^ mice after perfusion for 30 min with 1 µM ADX71441 (blue bar). Spike rate measured in individual neurons and averaged across a 10-min recording duration. (**D**) Pairwise correlations of spontaneous neuronal activity dependent on bin size. Standard error of the mean indicated as transparent bounds. At a bin size of 100 ms pairwise correlation in *Gabbr2*^I704N/+^ mice under ACSF perfusion was significantly increased compared to *Gabbr2*^+/+^ mice under ACSF perfusion and to *Gabbr2*^I704N/+^ mice under ADX71441 perfusion. (**E**) Distribution of the duration of synchronous events detected in the spontaneous population activity in **B** (see the ‘Materials and methods’ section). Under ACSF perfusion, the duration of synchronous events was significantly increased in *Gabbr2*^I704N/+^ compared to *Gabbr2^+/+^* mice (*top*). The duration of synchronous events in *Gabbr2*^I704N/+^ mice was significantly reduced under ADX71441 compared to ACSF perfusion (*bottom*). Statistical significance in **C** and **D** was evaluated using the Kruskal-Wallis test with Bonferroni multiple comparison correction, and in **E** using the Kruskal-Wallis test. *P < 0.05; ***P < 0.001; ^ns^*P* > 0.05. Box plot in **C** (MATLAB function boxchart): the central line is the sample median, the edges of the box are the 25th and 75th percentiles, whiskers extend to the most extreme data points not counting outliers, and the outliers are plotted as open circles.

To study the effects of ADX71441 on network activity, we conducted acute electrophysiological recordings in the primary auditory cortex (A1, layer II/III) of awake *Gabbr2*^I704N/+^ and *Gabbr2^+/+^* mice (Fig. 6A). Spontaneous cortical activity was recorded following a 30-min cortical surface perfusion, first with ACSF for baseline and then with ADX71441 (Fig. 6A). Sample recordings revealed that population neuronal activity in *Gabbr2^+/+^* and *Gabbr2*^I704N/+^ mice alternated between phases of increased synchronous activity and periods of low firing rates (Fig. 6B). Under baseline conditions, single-neuron firing rates, averaged over 10-min recordings, were significantly reduced in *Gabbr2*^I704N/+^ mice compared to *Gabbr2*^+/+^ mice (Fig. 6C). ADX71441 perfusion significantly increased spontaneous firing rates in *Gabbr2*^I704N/+^ neurons to levels comparable to *Gabbr2*^+/+^ mice under ACSF (Fig. 6B and C). At baseline, *Gabbr2*^I704N/+^ mice exhibited significantly higher pairwise correlation of neuronal activity compared to *Gabbr2*^+/+^ mice (Fig. 6D), indicating heightened network synchronization. Following ADX71441 perfusion, pairwise correlation levels in *Gabbr2*^I704N/+^ mice were significantly reduced, falling below those observed in *Gabbr2*^+/+^ mice (Fig. 6D). Similarly, the duration of synchronous events during spontaneous cortical activity, which was significantly prolonged in *Gabbr2*^I704N/+^ mice compared to *Gabbr2*^+/+^ mice, was normalized following ADX71441 application (Fig. 6E). Notably, previous studies have shown that GBR blockade enhances network synchronization.^63,64^ Thus, the heightened network synchronization in *Gabbr2*^I704N/+^ mice is consistent with weakened GBR signalling in response to synaptic GABA release.

**Figure 7:**
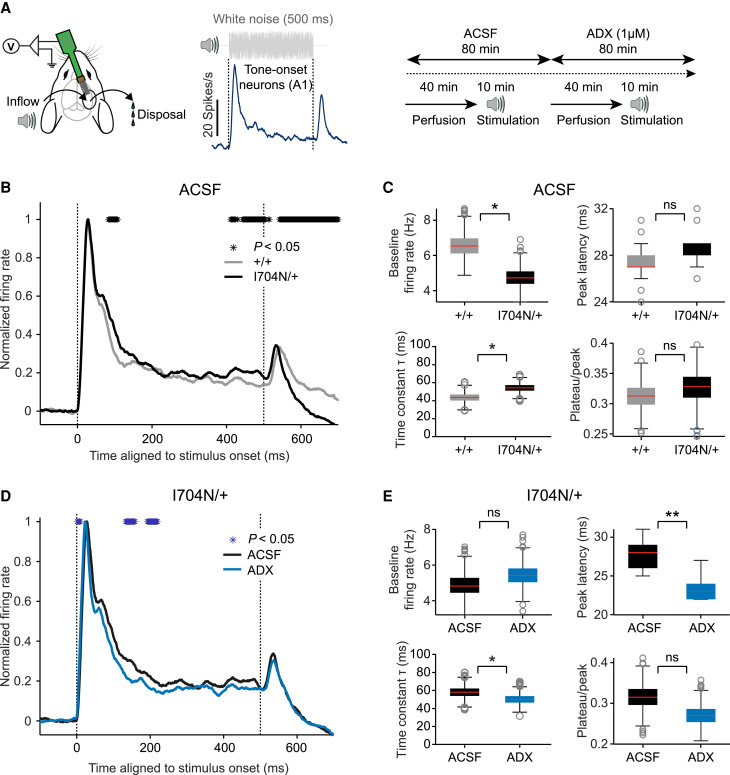
**ADX71441 normalizes response dynamics to sound stimulation in *Gabbr2*^I704N/+^ neurons.** (**A**) *Left*: In the multi-channel recording setup, a speaker is positioned 10 cm from the left ear. *Middle*: Example population peri-stimulus time histogram (PSTH) showing averaged neuronal responses to 500 ms white noise sound stimulation (80 dB), with 1 ms bins and a 20 ms sliding average for smoothing. *Right*: Schematic of the experimental timeline, with 10-min white noise stimulation sessions under artificial CSF (ACSF) and ADX71441 perfusion, beginning 40 min after the onset of ACSF perfusion. (**B**) Comparison of the response dynamics between *Gabbr2^+/+^* (*n* = 107 neurons, 3 mice, grey) and *Gabbr2*^I704N/+^ (*n* = 106 neurons, 3 mice, black) mice under ACSF perfusion. The *Gabbr2*^I704N/+^ neuronal population shows a slower firing decay rate of the tone-onset response and a saturation to higher firing rates. Averaged PSTHs were baseline-subtracted and normalized to the peak response. Time bins with significantly different firing rates (Wilcoxon rank sum test) between *Gabbr2^+/+^* and *Gabbr2*^I704N/+^ neurons are indicated with black stars. (**C**) Box plots show significantly reduced baseline firing rate and increased decay time constant τ in the *Gabbr2*^I704N/+^ neuronal population, compared to *Gabbr2^+/+^*. Baseline firing rate (*top left*) was measured during the 200 ms before stimulus onset. Peak latency (*top right*) was determined as the duration from stimulus onset to the peak of the population PSTH. τ (*bottom left*) was calculated by fitting an exponential decay function to the population PSTH from peak to stimulus offset. Plateau/peak (*bottom right*) was calculated as the ratio between the asymptote parameter derived from the exponential decay fit and the population peak. (**D**) Comparison of response dynamics between the *Gabbr2*^I704N/+^ neuronal population (*n* = 76 neurons, 3 mice) recorded under both ACSF (black) and ADX71441 (blue) perfusion indicates a faster firing rate decay following tone-onset response in the ADX71441 condition. Time bins with significantly different firing rates (Wilcoxon rank sum test) between ACSF and ADX71441 conditions are indicated with blue stars. (**E**) Box plots (values derived as in **C**) illustrate significantly reduced peak latency and decreased τ in *Gabbr2*^I704N/+^ neurons in the ADX71441 condition compared to the ACSF condition. Bootstrap resampling was used for data representation and significance testing in **C** and **E** (one-sided permutation test, 1000×, see the ‘Materials and methods’ section). *P < 0.05, **P < 0.01, ^ns^*P* > 0.05.

To examine the effects of ADX71441 on neuronal population dynamics in response to a sensory stimulus, we analysed transient sound-onset responses in the primary auditory cortex (A1), which encodes sound intensity and frequency.^65,66^ After a 40-min perfusion, first with ACSF and then with ADX71441, we stimulated the mice with 500 ms of white noise and analysed the population response of tone-onset neurons (Fig. 7A). As expected, tone-onset peak responses increased with sound intensity (40–80 dB; Supplementary Fig. 4A). Notably, the *Gabbr2*^I704N/+^ population response was broader and exhibited slower decay compared to the *Gabbr2*^+/+^ response (Fig. 7B and Supplementary Fig. 4A). However, the latency to the peak response did not differ between genotypes, while the baseline frequency in *Gabbr2*^I704N/+^ mice, measured during the 200 ms preceding stimulus onset, was significantly reduced compared to *Gabbr2*^+/+^ mice (Fig. 7C and Supplementary Fig. 4A). The timing of inhibitory inputs in A1 is delayed relative to excitatory inputs, contributing to the sharpening of spike timing.^66^ Consistent with this, blockade of GABA inhibition has been shown to result in slower population response decay.^67^ In line with a reduced GBR-mediated response to synaptic GABA, we observed a significantly slower decay—indicated by an increased time constant (τ)—of the population response in *Gabbr2*^I704N/+^ mice compared to *Gabbr2^+/+^* mice (Fig. 7C). The decay of the population response in *Gabbr2*^I704N/+^ mice was significantly faster in the presence of ADX71441, bringing τ closer to the value observed in *Gabbr2^+/+^* mice under ACSF (Fig. 7C and E; τ of *Gabbr2^+/+^* ACSF versus *Gabbr2*^I704N/+^ ADX, *P* = 0.1329). ADX71441 also accelerated the decay of the population response in *Gabbr2^+/+^* mice (Supplementary Fig. 4B and C). We further observed that in *Gabbr2*^I704N/+^ population responses, the latency from stimulus onset to the peak response was significantly shorter in the presence of ADX71441 (Fig. 7E), contributing to the sharpening of the response. The plateau/peak ratio of sound-onset responses was not affected in *Gabbr2*^I704N/+^ mice or by ADX71441 treatment (Fig. 7C and E and Supplementary Fig. 4C), supporting that the steady-state of the responses remains unchanged. In summary, we found reduced spontaneous firing rates, higher pairwise correlations of neuronal activity and prolonged spontaneous and evoked synchronous network activity in *Gabbr2*^I704N/+^ mice. ADX71441 had a normalizing effect on spontaneous network activity and sound-onset responses, supporting its potential for therapeutic use.

## Discussion

### Adaptive changes in response to constitutive GBR activity

Numerous *de novo* variants linked to neurodevelopmental, neurological and psychiatric disorders are found within the protein-coding regions of genes that encode synaptic proteins, including GBRs and their associated proteins.^17,18,34,68^ The mechanisms by which these variants disrupt synaptic functions often remain elusive. However, understanding these molecular mechanisms is essential for developing targeted therapeutic strategies.

The main *in vitro* pharmacological characteristic of the *GABBR2* missense variants analysed here was their strongly increased constitutive activity, observed both in homomeric GB2 subunits and heteromeric GB1/2 receptors. Structural data suggest that amino acid variations within the TMDs of these variants stabilize the active state of the GB2 subunit,^48^ thereby enabling G protein activation even in the absence of GABA. This increased constitutive activity simultaneously reduced agonist efficacy at the variant receptors, a phenomenon also observed with other GPCRs.^69^ As a result, the analysed variants exhibit a combination of gain- and loss-of-function phenotype, making it difficult to predict their impact on neuronal, synaptic and network activity without an appropriate animal model. To explore the pathogenic mechanism of a *GABBR2* variant with intermediate constitutive activity, we introduced the *GABBR2* p.I705N variation into the mouse *Gabbr2* gene. Our analysis revealed that *Gabbr2*^I704N/+^ and *Gabbr2*^I704N/I704N^ mice display an abnormal high-amplitude rhythm in the δ frequency band (1–4 Hz) in EEG recordings. *Gabbr2*^I704N/+^ mice showed significantly increased amplitudes during non-REM and REM sleep, while *Gabbr2*^I704N/I704N^ displayed elevated amplitudes during both sleep and wakefulness. The individual carrying the *GABBR2* p.I705N variant experienced infantile spasms accompanied by hypsarrhythmia on EEG.^19,20^ Recent PSD analyses of paediatric patients with hypsarrhythmia revealed an increase in δ frequency waves, similar as now seen in *Gabbr2*^I704N/+^ and *Gabbr2*^I704N/I704N^ mice.^70^ Likewise, high-amplitude δ waves in the EEG have been reported in individuals with Rett syndrome.^71^ Notably, the individual carrying the *GABBR2* p.I705N variant has remained largely seizure-free since the age of 5 years.^19^ *Gabbr2*^I704N/+^ and *Gabbr2*^I704N/I704N^ mice did not exhibit overt seizures, possibly because their EEGs were recorded at near-adult ages (8–22 weeks).

Electrophysiological recordings from hippocampal pyramidal neurons in both *Gabbr2*^I704N/+^ and *Gabbr2*^I704N/I704N^ mice revealed elevated tonic pre- and postsynaptic GBR activity, which is consistent with the increased constitutive GBR activity observed in the sreLuc assay *in vitro.* The significant reduction of tonic GBR activity observed in mutant mice upon treatment with an inverse agonist supports that this activity is largely driven by constitutively active heteromeric GB1/2 receptors, rather than homomeric GB2 subunits, which lack the binding site for the inverse agonist. Proteomic analysis of the brains from *Gabbr2*^I704N/+^ and *Gabbr2*^I704N/I704N^ mice revealed a significant reduction in the expression of GBR subunits, G protein subunits and other receptor signalling components, likely representing an adaptive response to the increased constitutive GBR activity. Although the mass spectrometry data do not allow us to determine whether the downregulation specifically affects wt or variant GB2 subunits (or both), the observed reduction in GB1 subunits and G protein levels is expected to impair signalling from both wt and variant receptors. Moreover, there was a significant upregulation of Kv1, Kv3 and Kir4 channels—none of which are direct effectors of GBRs. We propose that these K^+^ channels may compensate for the reduced activation of Kir3 channels resulting from impaired GBR signalling. Consistent with the disrupted GBR signalling, *Gabbr2*^I704N/+^ and *Gabbr2*^I704N/I704N^ mice also exhibited significantly reduced pre- and postsynaptic baclofen responses. In *Gabbr2*^I704N/+^ mice, which model individuals with a monoallelic *de novo GABBR2* variant, the reduced expression of GBR and G protein subunits likely causes a hypomorphic effect, impairing the signalling of GBRs encoded by the wt allele. Both *Gabbr2*^I704N/+^ and *Gabbr2*^I704N/I704N^ mice exhibit an impairment of LTP, similar to *Gabbr1*^−/−^ and *Gabbr1a*^−/−^ mice with a complete or partial loss of GBRs, respectively.^3,61,72^ A factor contributing to the impairment of LTP may be the reduced GBR signalling at glutamatergic terminals, resulting in LTP saturation due to excess glutamate release.^61^ Similarly, reduced GBR signalling at GABA terminals may impair LTP by increasing postsynaptic inhibition, resulting from excessive GABA release.^3,62,72^ The impairment of LTP observed in the mice may contribute to the severe intellectual disability seen in individuals with DEE59.^19^

A previous study showed that constitutively active GBR variants exhibit reduced neuronal surface expression, leading to decreased signalling efficacy.^24^ Our results extend these findings by demonstrating that the impaired GABA-induced receptor signalling observed in the brain arises not only from reduced surface expression, but also from the diminished responsiveness of constitutively active receptors to GABA, as well as from a compensatory downregulation of total GBR protein levels and associated signalling components. Both the *in vitro* and slice electrophysiology data from *Gabbr2*^I704N/+^ brain slices do not support that GB2 variants signal from intracellular compartments. Impaired GBR signalling as a consequence of constitutive activity provides an explanation for the clinical similarities between constitutively active *GABBR2* variants and loss-of-function *GABBR1* variants.^17^

### Therapeutic approaches to increase synaptic GBR inhibition

Several GPCR variants exhibiting constitutive activity have been implicated in human disorders.^73^ Inverse agonists are commonly used to treat such conditions by suppressing basal GPCR activity.^74^ However, our findings indicate that a constitutively active *GABBR2* variant leads to reduced pre- and postsynaptic GBR responses to GABA *in vivo*. This reduction appears to increase the E/I ratio within neuronal networks and gives rise to clinical features that overlap with those caused by loss-of-function *GABBR1* variants.^17^ Given this pathophysiology, inverse agonists—which further reduce GBR signalling—could exacerbate the condition. Similarly, chronic baclofen administration may trigger compensatory downregulation of receptor components, resulting in an additional loss of function. This is supported not only by our proteomic data but also by previous reports that chronic baclofen treatment often leads to receptor desensitization and the development of tolerance.^59^ In contrast, PAMs that potentiate GABA responses at wt GBRs in patients may offer greater therapeutic benefit. PAMs act presynaptically to reduce neuronal excitability by suppressing glutamate release, as shown in *Gabbr2*^I704N/+^ brain slices and in transfected neurons overexpressing the *GABBR2* p.S695I variant.^24^ They also enhance postsynaptic inhibitory signalling by increasing GBR-mediated K⁺ currents.^75^ To evaluate the therapeutic potential of a PAM in *Gabbr2*^I704N/+^ mice, we selected ADX71441, the most advanced clinical candidate available at the time. We reasoned that, pending successful clinical trial outcomes, individuals carrying pathogenic *GABBR1* or *GABBR2* variants could potentially benefit from treatment with ADX71441 or structurally related follow-up compounds. Ideally, such compounds should lack ago-PAM activity to avoid downregulation of receptor signalling. ADX71441 has been reported to display low ago-PAM activity in *in vitro* assays,^35^ and similarly showed minimal intrinsic activity at wt receptors in our sreLuc assay, which—being an accumulation assay—is particularly sensitive to weak PAM activity. ADX71441 is orally bioavailable, brain-penetrant and exhibits a well-characterized pharmacokinetic and pharmacodynamic profile.^35,76^ Importantly, it has been shown to induce less tolerance upon chronic administration and to cause fewer side effects—such as muscle relaxation—than baclofen.^77,78^ In our study, ADX71441 normalized spontaneous firing rates and restored both spontaneous and sound-evoked synchronous activity in the auditory cortex of awake *Gabbr2*^I704N/+^ mice. These results suggest that PAMs may represent a viable therapeutic strategy for patients with constitutively active *GABBR2* variants, as well as *GABBR1* loss-of-function variants.

## Supplementary Material

awaf356_Supplementary_Data

## Data Availability

The data that support the findings of this study are available from the corresponding author, upon reasonable request.
