# Supplementary material for: Normalization of network activity in an epilepsy model with a constitutively active GABBR2 variant
Source: Brain. 2025 Sep 25;149(6):2166–83. doi: 10.1093/brain/awaf356 (PMC13232046; doi:10.1093/brain/awaf356)
Supplement: awaf356_Supplementary_Data [file awaf356_Supplementary_Data.zip › brain-2025-00444-File010.pdf]

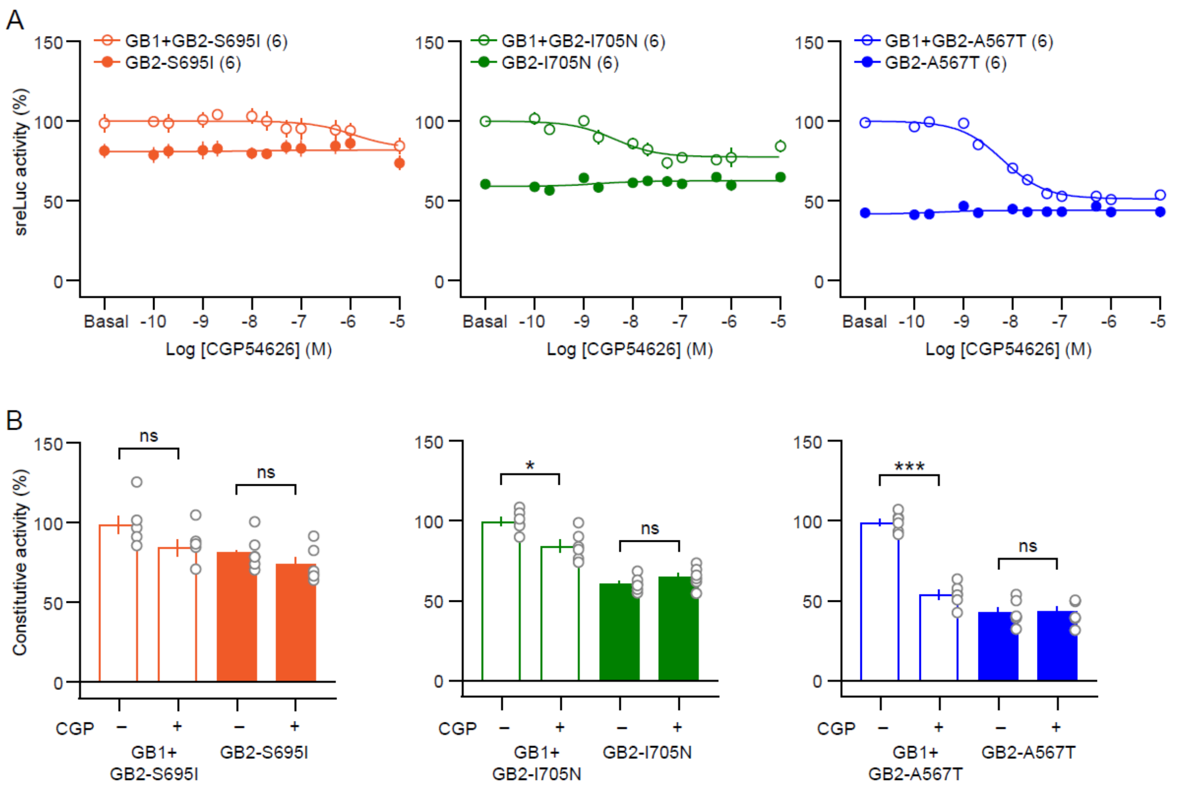

Figure S1. **The inverse agonist CGP54626 does not inhibit constitutive activity of homomeric GB2-S695I, GB2-I705N and GB2-A567T.** (**A**) Inhibition curves for the inverse agonist CGP54626, which binds to the orthosteric binding-site on GB1, using the sreLuc assay in HEK293 cells expressing GB2 variants alone (closed circles) or together with GB1 (open circles). SreLuc activity was normalized to the constitutive activity of each variant co-expressed with GB1 at baseline The number of independent experiments is indicated. (**B**) Quantification of constitutive activity from the inhibition curves in (**A**) at baseline (-) and at 10μM CGP54626 (+). CGP54626 does not significantly inhibit constitutive activity of homomeric GB2 variants. All data are mean ± SEM. Paired t-test. *** p<0.001, * p<0.05, ns p>0.05.

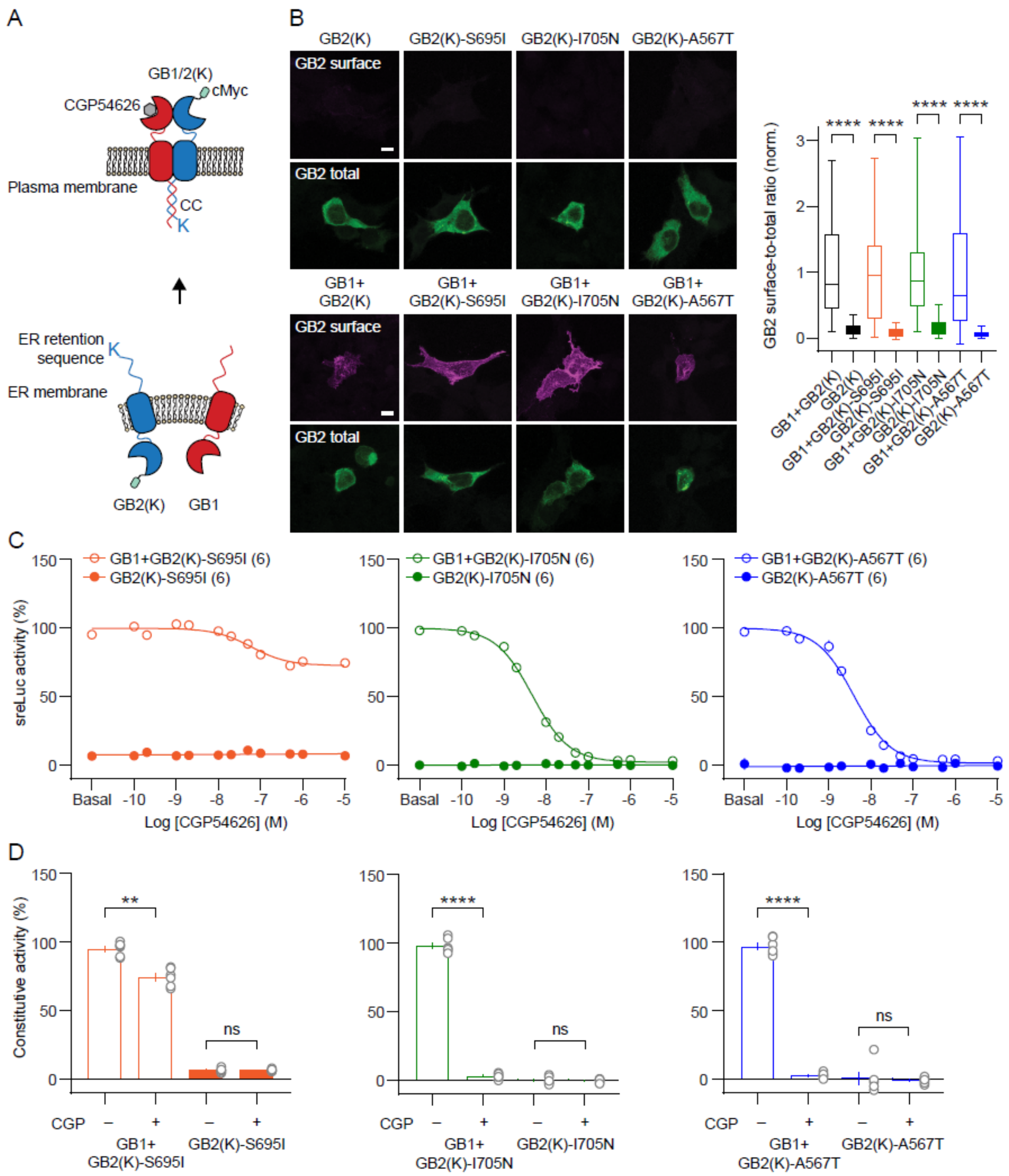

Figure S2. The inverse agonist CGP54626 fully inhibits constitutive activity of heteromeric receptors assembled with GB2-I705N and GB2-A567T, but not with GB2-S695I. (**A**) Approach used to limit GB2 surface expression to heterodimers.[36] The C-terminal lysine-based ER retention signal KKTN was inserted into the C-terminal domain of Myc-GB2, generating Myc-GB2(K) subunits that are retained in the ER in the absence of GB1.[36] Coiled-coil (cc) interactions with GB1 mask the retention signal, enabling GB1/GB2(K) heteromers to

reach the cell surface. (**B**) GB2(K) subunits selectively traffic to the cell surface upon heteromerization with GB1. Cell surface and total Myc-GB2(K) expression levels of GB2(K) subunits expressed alone (top) or in combination with GB1 (bottom) in HEK293T cells. Surface GB2(K) immunofluorescence was assessed in living cells using an anti-Myc antibody. Total GB2(K) immunofluorescence was determined with an anti-GB2 antibody after fixation and permeabilization of the cells. Scale bar: 10 µm. The box plot shows GB2(K) surface-to-total ratios for GB2(K) (n=76 cells), GB2(K)-S695I (n=53), GB2(K)-I705N (n=73), and GB2(K)-A567T (n=55) subunits as well as co-expression of GB1+GB2(K) (n=76 cells), GB1+GB2(K)-S695 (n=63), GB1+GB2(K)-I705N (n=90), and GB1+GB2(K)-A567T (n=93). Data were normalized to the GB2(K) surface-to-total expression ratio of GB1+GB2(K). (**C**). Inhibition curves for the inverse agonist CGP54626, which binds to the orthosteric GABA binding-site on GB1, were generated using the sreLuc assay. When co-expressed with GB1 (open circles), CGP54626 completely inhibits the constitutive activity of receptors containing GB2(K)-I705N and GB2(K)-A567T, and partially inhibits those with GB2(K)-S695I. Due to ER retention, GB2(K) variants expressed alone do not exhibit constitutive activity (closed circles). SreLuc activity was normalized to the constitutive activity of heteromeric receptors at baseline. The number of independent experiments is indicated. (**D**) Quantification of constitutive activity from the experiments in panel (**C**) at baseline (-) and at 10 µM CGP54626 (+). Data are shown as box plot (median, interquartile range, minimum, maximum) or mean ± SEM. Kruskal-Wallis with Dunn's multiple post-hoc tests (**B**) and paired t-test (**D**). **** $p<0.0001$, ** $p<0.01$, ns $p>0.05$.

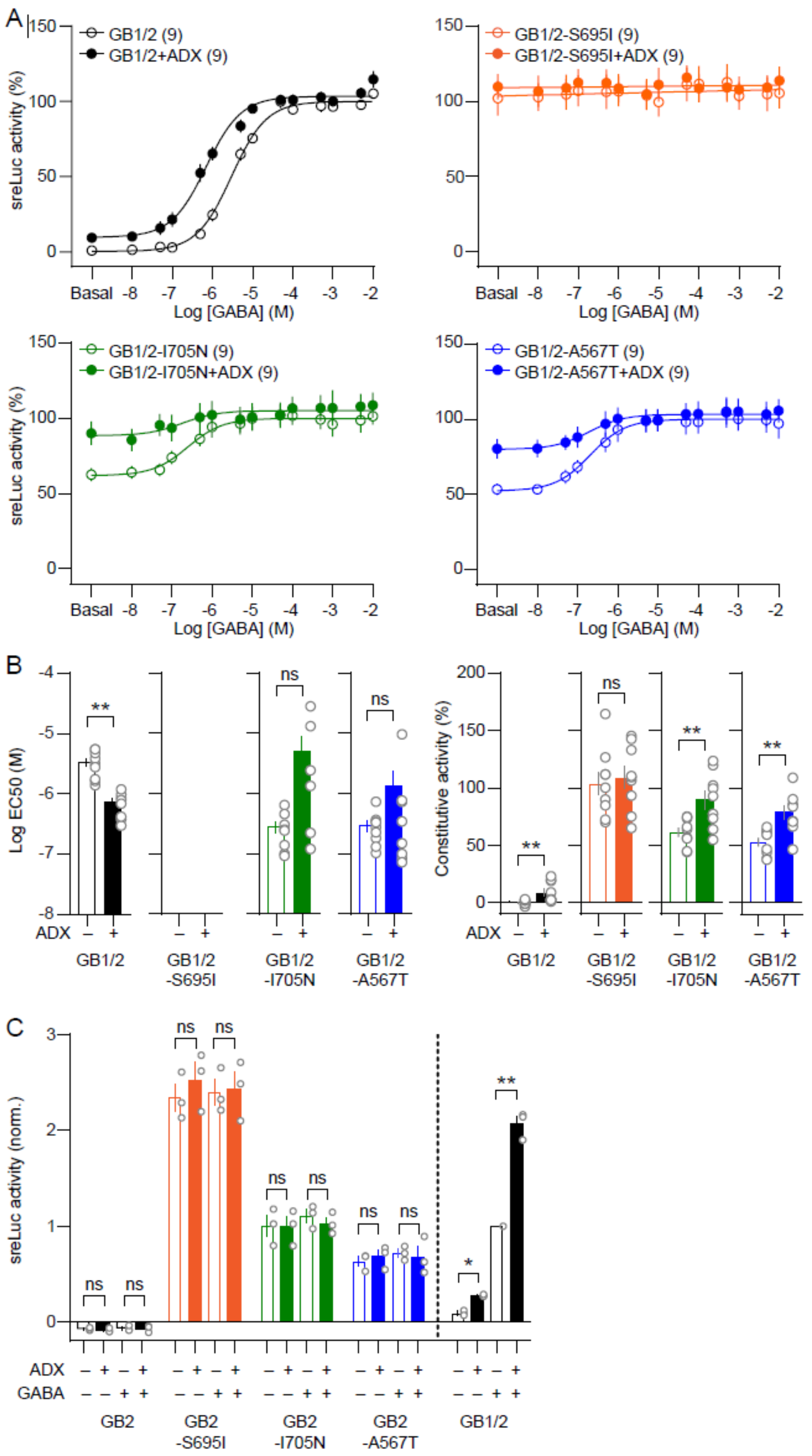

Figure S3. Allosteric agonism of the PAM ADX71441 at GBR variants. (A) Concentration-response curves of GABA-induced sreLuc activity at wt and variant GBRs expressed in HEK293T cells, in the absence and presence of 20 nM ADX71441. Data are normalized to the

maximal sreLuc activity in the absence of ADX71441. The number of independent experiments is indicated. (**B**) EC_{50} values and constitutive activity in the absence and presence of ADX71441 from the experiments in panel (**A**). (**C**) SreLuc activity of wt and variant GB2 subunits in the absence and presence of 20 nM ADX71441 and/or 1 μM GABA. Data were normalized to the sreLuc activity of wt GBRs (GB1/2) in the presence of 1 μM GABA (n=3 independent experiments). All data are presented as mean ± SEM. Wilcoxon matched-pairs signed rank test (**B**) and paired t-test (**C**). ** $p<0.01$, * $p<0.05$, ns $p>0.05$.

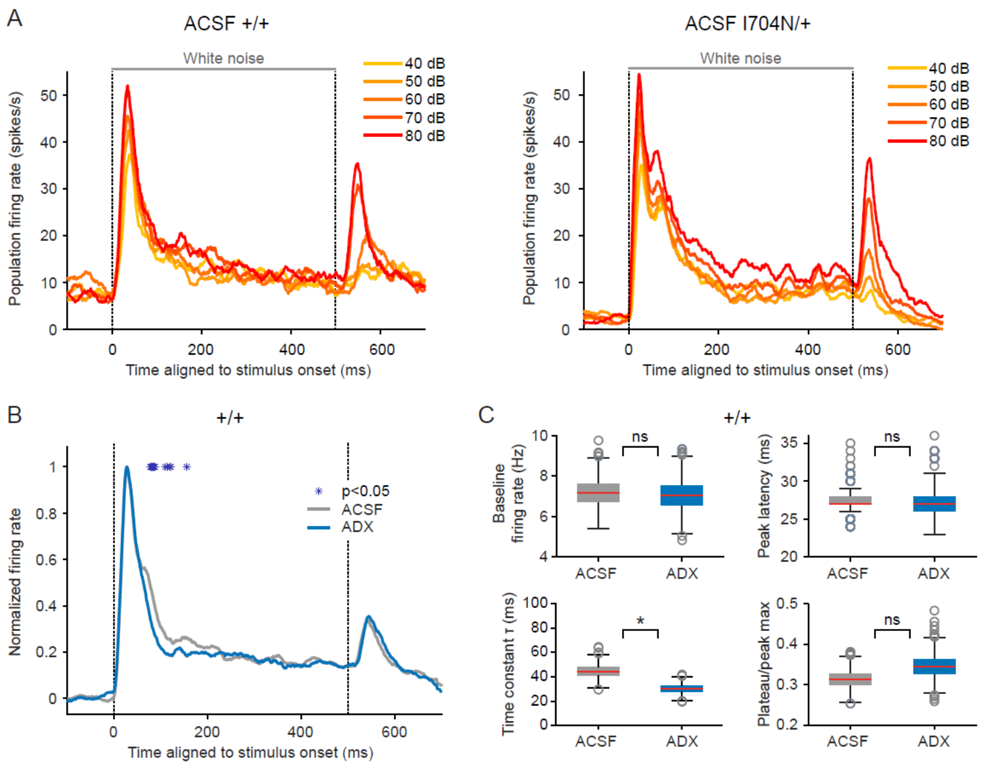

Figure S4. Auditory responses in *Gabbr2*$^{+/+}$ and *Gabbr2*$^{I704N/+}$ mice. (A) Population peri-stimulus time histograms (PSTHs) from tone-onset responsive neurons in the primary auditory cortex of *Gabbr2*$^{+/+}$ and *Gabbr2*$^{I704N/+}$ mice in response to white noise stimulation at increasing levels (40-80 dB) for 500 ms. The results represent averages from a single recording session following 30 minutes ACSF perfusion (*Gabbr2*$^{+/+}$, n= 27 neurons; *Gabbr2*$^{I704N/+}$, n=33 neurons). PSTHs were binned into 1 ms intervals and smoothened with a 20 ms sliding average. (**B**) Comparison of response dynamics between the *Gabbr2*$^{+/+}$ neuronal populations (n=86 neurons, 3 mice) recorded under both ACSF (grey) and ADX71441 (blue) perfusion indicates a faster firing rate decay following tone-onset response in the ADX71441 condition. Averaged PSTHs were baseline-subtracted and normalized to the peak response. Time bins with significantly different firing rates (Wilcoxon rank sum test) between ACSF and ADX71441 condition indicated with blue stars. **(C)** Box plots (median, interquartile range, minimum, maximum) illustrate significantly decreased exponential decay time constant (τ) in the ADX71441 as compared to the ACSF condition (values derived as described in Figure 7C). * $p<0.05$, ns $p>0.05$.
