# Supplementary figures and images for: Normalization of network activity in an epilepsy model with a constitutively active GABBR2 variant
Source: Brain. 2025 Sep 25;149(6):2166–83. doi: 10.1093/brain/awaf356 (PMC13232046; doi:10.1093/brain/awaf356)

# Full-length Western blots of Figure 4C

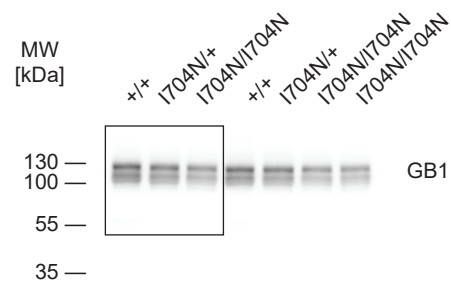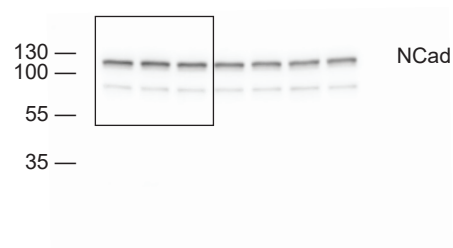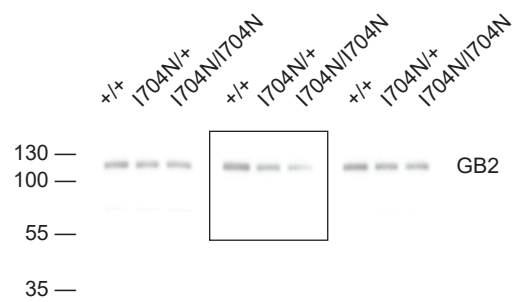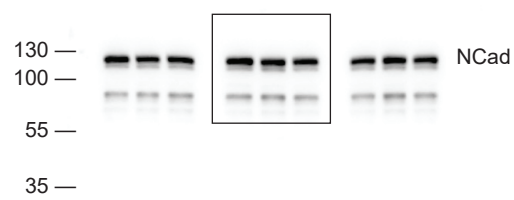

Supplement: awaf356_Supplementary_Data [file awaf356_Supplementary_Data.zip › brain-2025-00444-File009.pdf]
